# Efficacy of Tibial Nerve Stimulation in Neurogenic Lower Urinary Tract Dysfunction Among Patients with Multiple Sclerosis: A Systematic Review and Meta-analysis

**DOI:** 10.5152/tud.2023.22241

**Published:** 2023-03-01

**Authors:** Fateme Tahmasbi, Samaneh Hosseini, Sakineh Hajebrahimi, Reza Mosaddeghi Heris, Hanieh Salehi-Pourmehr

**Affiliations:** 1 Student Research Committee, Tabriz University of Medical Sciences, Tabriz, Iran; 2 Research Center for Evidence-based Medicine, Iranian EBM Centre: A Joanna Briggs Institute (JBI) Center of Excellence, Tabriz University of Medical Sciences, Tabriz, Iran; 3 Neurosciences Research Center, Tabriz University of Medical Sciences, Tabriz, Iran

**Keywords:** Tibial nerve stimulation, multiple sclerosis, neurogenic lower urinary tract dysfunction, neurogenic bladder, overactive bladder, systematic review

## Abstract

**Objective:**

This study was performed to systematically review the current literature on the effects of transcutaneous tibial nerve stimulation and percutaneous tibial nerve stimulation on multiple sclerosis-induced neurogenic lower urinary tract dysfunction.

**Materials and methods:**

Medical databases including PubMed, Scopus, Embase, and Web of Science were systematically searched from inception to September 2022. Meta-analysis was carried out using the comprehensive meta-analysis tool.

**Results:**

Our inclusion criteria were met by 12 studies evaluating the effects of percutaneous tibial nerve stimulation/transcutaneous tibial nerve stimulation on multiple sclerosis-induced neurogenic lower urinary tract dysfunction. Comparing the post-intervention results to the baseline showed that the rate of frequency was decreased in both percutaneous tibial nerve stimulation and transcutaneous tibial nerve stimulation groups after intervention. The overall mean change of tibial nerve stimulation on frequency was –2.623 (95% CI: –3.58, –1.66; *P* < .001, *I* ^2^: 87.04) among 6 eligible studies. The post-void residual was decreased after treatment in both methods of tibial nerve stimulation, with an overall mean difference of –31.13 mL (95% CI: –50.62, –11.63; *P* = .002, *I* ^2^: 71.81). The other urinary parameters, including urgency (mean difference: –4.69; 95% CI: –7.64, –1.74; *P* < .001, *I* ^2^: 92.16), maximum cystometric capacity (mean difference: 70.95; 95% CI: 44.69, 97.21; *P* < .001, *I* ^2^: 89.04), and nocturia (mean difference: –1.41; 95% CI: –2.22, 0.60; *P* < .001, *I* ^2^: 95.15), were improved after intervention, too. However, the results of subgroup analysis showed no effect of transcutaneous tibial nerve stimulation on urinary incontinence (mean difference: –2.00; 95% CI: –4.06, 0.06; *P* = .057, *I* ^2^: 95.22) and nocturia (mean difference: –0.39; 95% CI: –1.15, 0.37; *P* = .315, *I* ^2^: 84.01). In terms of mean voided volume, the evidence was related to only percutaneous tibial nerve stimulation with a mean change of 75.01 mL (95% CI: –39.40, 110.61; *P* < .001, *I* ^2^: 85.04).

**Conclusion:**

Although the current literature suggests that tibial nerve electrostimulation might be an effective method for treating neurogenic lower urinary tract dysfunction, the evidence base is poor and derived from small, mostly nonrandomized trials with a high risk of bias and confounding.

**Main Points**

- One of the most prevalent problems among multiple sclerosis (MS) patients is neurogenic lower urinary tract dysfunction (NLUTD), for which there is now no definite treatment.
- Two of the most promising neuromodulation techniques for minimizing MS-induced NLUTD are transcutaneous tibial nerve stimulation and percutaneous tibial nerve stimulation which are the focus of this systematic review.
- The meta-analysis of 8 studies found that individuals with MS-related NLUTD may benefit from percutaneous or transcutaneous electrostimulation of the tibial nerve.
- Despite offering level 1 evidence, generalizability of the findings of this study is limited, especially as a result of the inclusion of uncontrolled, nonrandomized trials with small populations.

## Introduction

Neurogenic lower urinary tract dysfunction (NLUTD) is one of the most frequent complaints in multiple sclerosis (MS) patients. Approximately 90% of MS patients experience urologic symptoms 10 years after the outbreak of the disease, while 5%-10% of patients have bladder disturbances at the beginning of the disease.^1,2^

Considering their troublesome nature, these symptoms can severely affect patients’ quality of life (QoL).^3^ The bladder dysfunction can be attributed to several pathophysiological pathways, including impulse blockage in demyelinated axons, conduction failure due to neuronal degeneration, and possible functional impairment of cytokines.^4^

The first and the second pathology are related to damage in bladder control and the third pathology links the bladder disturbances to the dysfunction of receptors and neurotransmitters which are responsible for bladder control. In MS, NLUTD occurs as a consequence of spinal cord involvement above the sacral segment, leading to urinary symptoms including increased frequency and urgency of micturition, nocturia, incontinence, and inability to empty the bladder completely. The first 2 are suggested to be the most frequent ones.^5,6^

Approaching the MS-induced NLUTD consists of a multidisciplinary method. For instance, intermittent self-catheterization offers one of the best methods of coping with incomplete bladder emptying and urinary retention. Medications including antimuscarinics benefit patients with frequency, nocturia, urgency, or urge incontinence.^7^

Other approaches are available in cases where antimuscarinics are ineffective or poorly tolerated, including intradetrusor botulinum toxin, or nerve stimulation methods including tibial nerve stimulation (TNS) and sacral neuromodulation.^8-10^

Neuromodulation is, as defined by the International Neuromodulation Society, the use of implantable and non-implantable electrical or chemical technologies to enhance the quality of human life and functioning. The use of neuromodulation has been increased recently, especially for managing chronic pain, musculoskeletal disorders, psychiatric disorders, and epilepsy.^10^

Transcutaneous tibial nerve stimulation (TTNS) and percutaneous tibial nerve stimulation (PTNS) are 2 types of neuromodulation that have been proposed for the treatment of MS-related urinary disturbances.^11-13^ These techniques rely on electrical stimulation of the tibial nerve to constrain the detrusor muscle. The most frequently reported intervention in the greater part of academic studies consists of 30-minute stimulation sessions performed every week for 10-12 weeks.^14^

Having the importance of managing NLUTD and its high prevalence among MS patients in mind, this study aims to systematically review the current literature on the effects of TNS (PTNS/TTNS) on the MS-induced NLUTD.

## Materials and Methods

The Preferred Reporting Items for Systematic Reviews and Meta-Analyses (PRISMA) Statement was followed when conducting this study to ensure accurate data reporting.^15^ The protocol was registered in the International Prospective Register of Systematic Reviews under the code CRD42022360571.

### Information Sources and Search Strategy

From their creation to September 2022, a number of medical databases, including PubMed, Scopus, Embase, and Web of Science, were thoroughly searched. They were then updated in October 2021. Google Scholar and all the references of the included studies were also checked for items that met the inclusion criteria, and those were imported to make sure there was complete saturation. The main search terms were as follows: “multiple sclerosis,” “MS,” “tibial nerve stimulation,” “percutaneous electric nerve stimulation,”, “PTNS,” “transcutaneous electrical nerve stimulation,” “TTNS,” “neuromodulation,” “neurogenic bladder,” “urinary bladder,” “overactive bladder,” “urinary incontinence,” and “neurogenic lower urinary tract dysfunction.” The Endnote X20 citation manager software was used to import the search results for further exploration.

### Eligibility Requirements

Two impartial reviewers conducted the eligibility evaluation (F.T. and S.H.). A third reviewer was consulted to settle any disagreements (H.S.). Studies were selected for further survey if they met all of the following criteria: (1) studies aiming to determine the effects of PTNS and/or TTNS on NLUTD in MS patients; (2) a population consisting of humans; and (3) available English full text.

Unoriginal articles including any type of reviews, conference proceedings, letters, and commentaries were excluded.

### Quality Assessment

The quality of included studies was assessed through the Joanna Briggs Institute tools of critical appraisal, each individualized based on the methodology of the study.^16^ In cases of disagreement, a third reviewer evaluated the study for confirmation after 2 authors independently evaluated the quality of the studies.

### Data Collection Process and Data Items

In a predetermined Excel sheet, 2 authors (F.T. and H.S.) extracted the data from the included studies. From each of the included studies, the following information was taken: data on citations including the first author’s name; the publication’s year and place of publication; the number, condition, and age of patients; the condition of the control group; the type of intervention used; the number, length, and frequency of therapy sessions; the length of the follow-up period; and the main outcomes of measure.

### Synthesis of Results

The comprehensive meta-analysis tool v3.7z was used to conduct the meta-analysis. For identifying heterogeneity within the studies, the *Q* statistic was used. Additionally, the *I* ^2^ statistic was used to calculate the effect of study heterogeneity. Low *I* ^2^ was defined as 25%, moderate as 25%-75%, and high as >75%. A fixed-effect model was used when there was no statistically significant difference in the heterogeneity (*P* < .05); otherwise a random effect model was applied.

## Results

### Literature Search and Description of Studies

**Figure 1.**
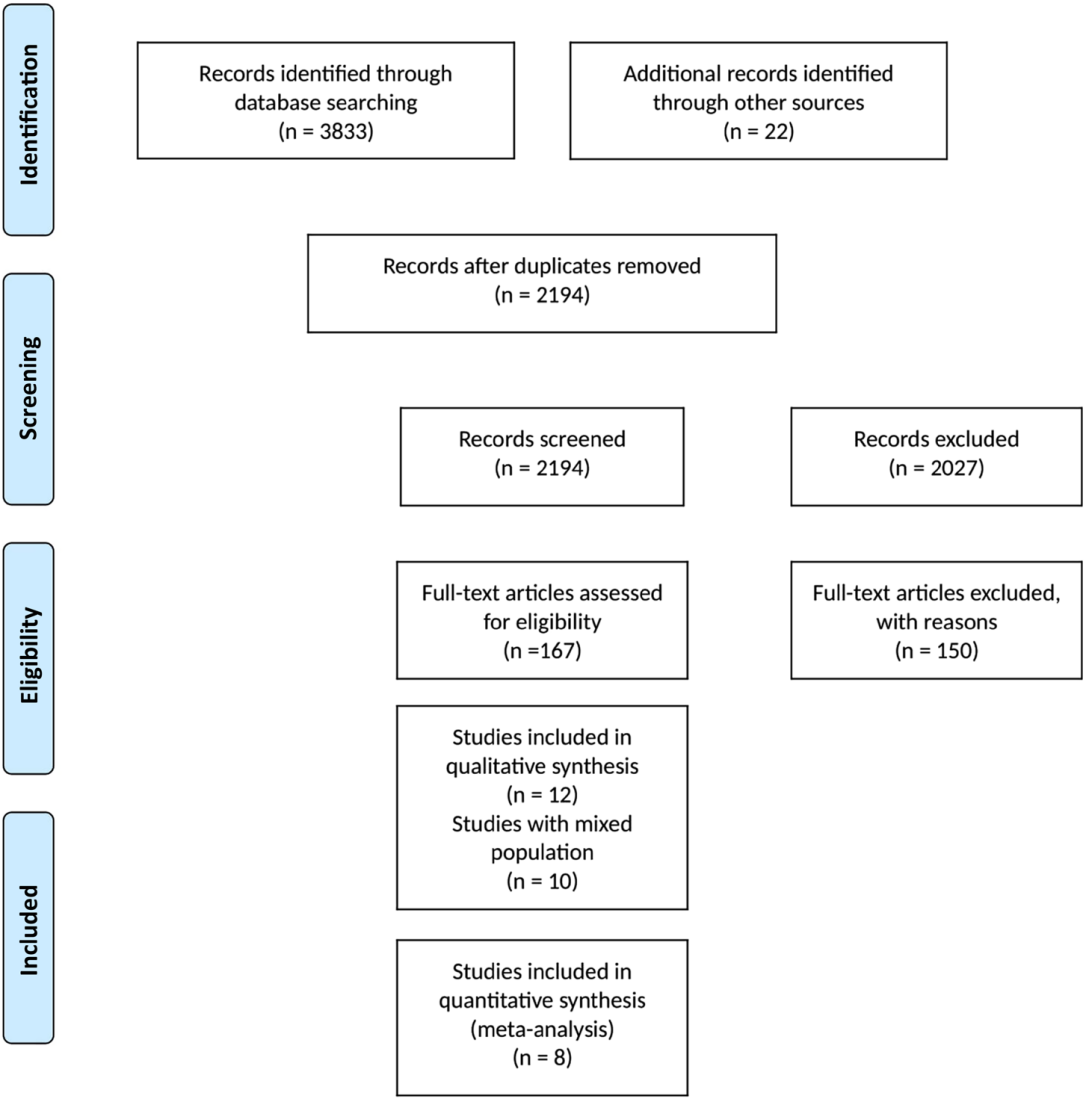
The Preferred Reporting Items for Systematic Reviews and Meta-Analyses flow diagram representing the selection process.

Up until September 2022, we discovered 3855 publications through the use of electronic databases, manual searches, and reference checking. After the duplicate studies were eliminated, 2194 studies underwent title/abstract screening. After reviewing the full texts of 41 articles, we determined that 12 studies satisfied our inclusion criteria and were included in this systematic review. A total of 8 studies were eligible for meta-analysis. In addition, 10 studies were found with mixed population; however, they did not separate data for the MS patients; therefore, we were unable to analyze their findings (Supplementary Table 1). The PRISMA flow diagram displays more details about the selection procedure (Figure 1).

### Summary of the Evidence

Tables 1 and 2 show the characteristics and the quality of the included studies, respectively. Twelve clinical studies were included, which assessed the outcomes of TNS among MS population.

Seven studies used PTNS^17-23^ and the other 5 used TTNS as the neuromodulation technique.^24-28^ Five studies took place in Turkey,^18-20,26,25^ 2 studies in the United Kingdom,^22,23^ and 1 in each of the following countries: Denmark,^17^ Switzerland,^21^ France,^24^ and Bosnia and Herzegovina.^28^ Three studies had a parallel control group—2 was a nonrandomized clinical trial with a control group consisting of pelvic floor muscle training^25,26^ and the other 1 was a randomized clinical trial with a control group receiving 5 mg oxybutynin tablet twice a day for 3 months.^28^ The Expanded Disability Status Scale score was reported in 9 studies and varied from the minimum of 3.40 to 4.8.

Furthermore, publications with mixed populations were reviewed to check for possible MS inclusions. Ten articles were found with a population consisting of patients with overactive bladder (OAB) symptoms, among which some were diagnosed with MS.^29-38^ In 5 articles TTNS and in the other 5 PTNS were sued as the intervention. All of these publications, except for 1, supported the beneficial effects of TNS in enhancing different parameters regarding the OAB symptoms. However, none of these articles reported data exclusive for MS patients; therefore, they are taken into consideration in our study. The results are presented in Supplementary Table 1.

We assessed the overall outcomes of the daily voiding frequency, daily leakage episodes (incontinence), urgency episodes, frequency of nighttime urination (nocturia), and cystometric parameters including mean voiding volume (MVV), maximum cystometric capacity (MCC), and post-void residual (PVR). The overall analysis results are presented in Table 3.

### Meta-analysis of Daily Voiding Frequency

**Figure 2.**
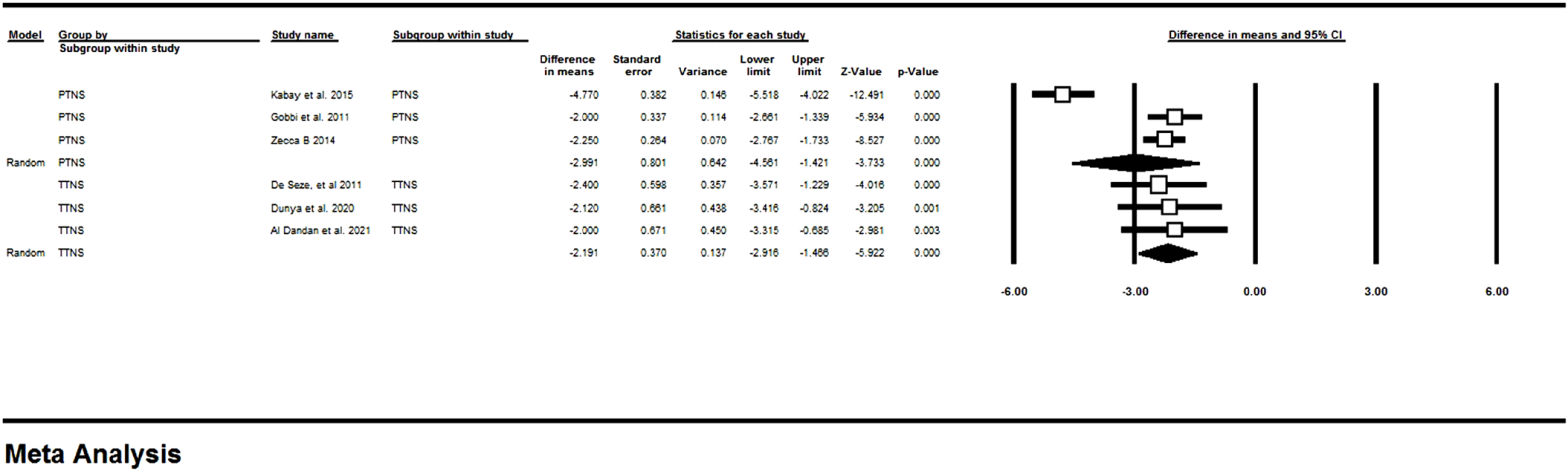
Meta-analysis of the daily voiding frequency. PTNS, percutaneous tibial nerve stimulation; TTNS, transcutaneous tibial nerve stimulation.

Figure 2 shows the results of meta-analysis of the pre- and post-intervention values of 6 studies (3 PTNS and 3 TTNS) regarding the daily frequency. Accordingly, the frequency was decreased significantly after the stimulation of tibial nerve (MD: –2.62, 95% CI: –3.58 to –1.66 and *P* < .001; *I* ^2^: 87.04). The subgroup analysis of showed that PTNS (MD: –2.99, 95% CI: –4.56 to –1.42, and *P* < .001; *I* ^2^: 94.52) and TTNS (MD: –2.19, 95% CI: –2.91 to –1.46, and *P* < .001; *I* ^2^: 0) each can significantly reduce the frequency as well.

### Meta-analysis of Daily Leakage Episodes

**Figure 3.**
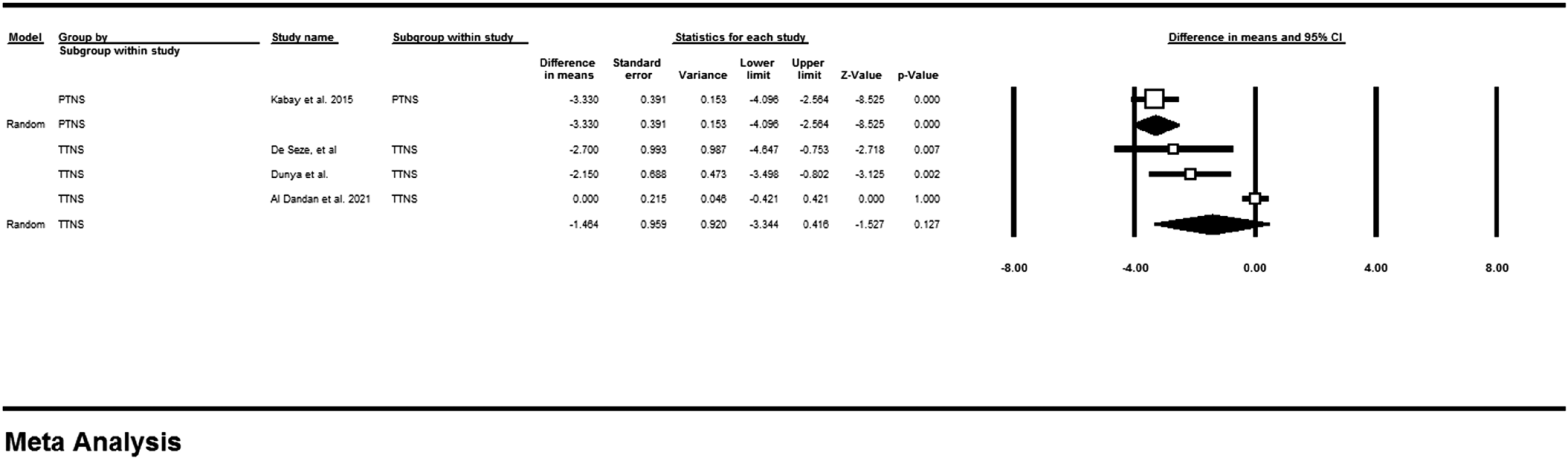
Meta-analysis of the daily leakage episodes. PTNS, percutaneous tibial nerve stimulation; TTNS, transcutaneous tibial nerve stimulation.

Daily leakage or incontinence episode was reduced after the application of PTNS in 1 study (MD: –3.33, 95% CI: –4.09 to –2.56, and *P* < .001; *I* ^2^: 0). The TTNS subgroup and the overall effect of TNS on urinary incontinence episodes did not show a significant reduction (MD: –1.46, 95% CI: –1.52 to 0.127, and *P* = .127), and MD: –2.00 (95% CI: –4.06 to 0.060, and *P* = .057). The results of meta-analysis are shown in Figure 3.

### Meta-analysis of Nocturia

**Figure 4.**
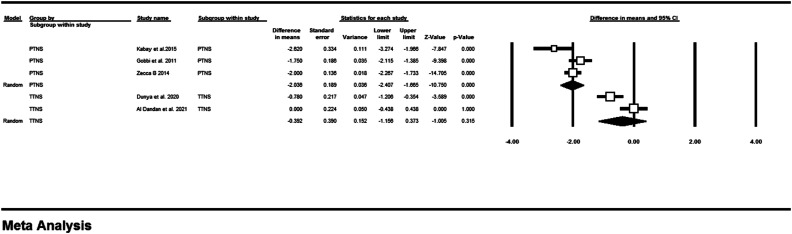
Meta-analysis of the nocturia. PTNS, percutaneous tibial nerve stimulation; TTNS, transcutaneous tibial nerve stimulation.

The meta-analysis of 6 studies, presented in Figure 4, showed that nocturia episodes are decreased significantly following TNS (MD: –1.41, 95% CI: –2.22 to –0.60, and *P* < .001). Percutaneous tibial nerve stimulation and TTNS subgroups each also reduced these episodes; however, the reduction was statistically significant in PTNS (MD: –2.03, 95% CI: –2.40 to –1.66, and *P* < .001), while it was insignificant in TTNS (MD = –0.39, 95% CI: –1.005 to 0.315 and *P* = .315).

### Meta-analysis of Urgency Episodes

**Figure 5.**
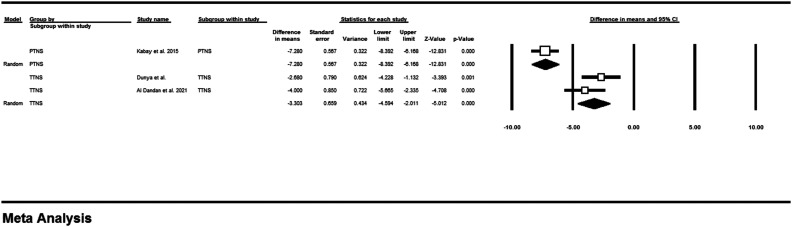
Meta-analysis of the urgency episodes. PTNS, percutaneous tibial nerve stimulation; TTNS, transcutaneous tibial nerve stimulation.

As presented in Figure 5, daily urgency episodes, experienced by patients from 3 studies, were decreased significantly (MD: –4.69, 95% CI: –1.504 to –7.64, and *P* < .001).

### Cystometric Parameters

**Figure 6.**
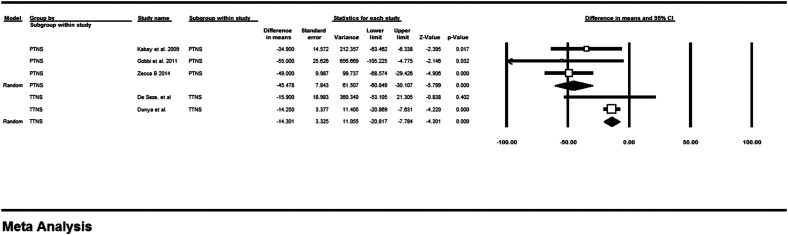
Meta-analysis of the post-voiding residual. PTNS, percutaneous tibial nerve stimulation; TTNS, transcutaneous tibial nerve stimulation.

**Figure 7.**
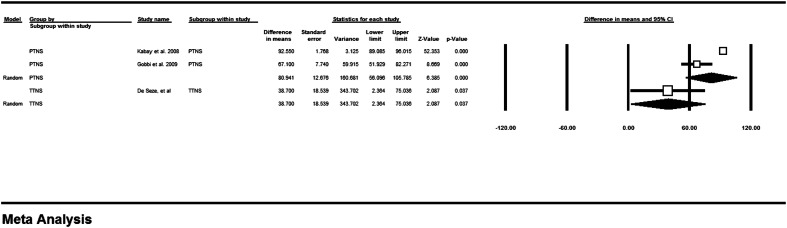
Meta-analysis of the maximum cystometric capacity. PTNS, percutaneous tibial nerve stimulation; TTNS, transcutaneous tibial nerve stimulation.

**Figure 8.**
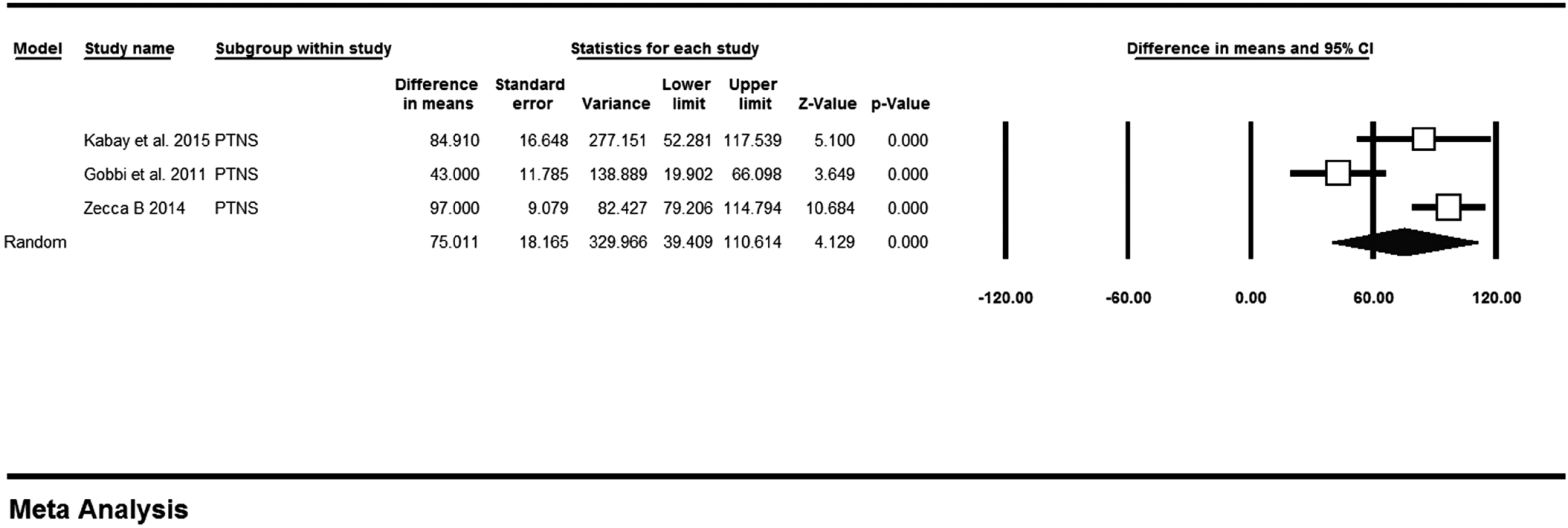
Meta-analysis of the mean voiding volume. PTNS, percutaneous tibial nerve stimulation.

The results of meta-analyzing PVR, MCC, and MVV are presented in Figures 6, 7, and 8, respectively. According to the meta-analysis of 5 studies, PVR shows a significant reduction after the application of TNS (MD: –31.13, 95% CI: –50.62 to –11.63, and *P* = .002). The subgroup analysis shows that PTNS and TTNS showed a significant reduction in PVR (MD: –45.47, 95% CI: –60.84 to –30.10, and *P* < .001; MD: –14.30, 95% CI: –20.81 to –7.78, and *P* < .001, respectively).

In 3 studies, MCC was insignificantly enhanced (MD: 70.95, 95% CI: 44.69 to 97.21, and *P* < .001) and MVV was significantly increased after only in PTNS eligible studies (MD: 75.01, 95% CI: 39.40 to 110.61, and *P* < .00) (Figures 7 and 8, respectively).

## Discussion

The aim of our systematic review was to analyze the scientific evidence on the treatment of MS-induced NLUTD through PTNS or TTNS procedures and evaluate the results of post-intervention with baseline amounts. Tibial nerve electrostimulation appears to be promising interventions, according to the results of our review.

Multiple sclerosis is a unique neurological disease. It manifests with a broad spectrum of clinical presentations. These symptoms are related with time and disease course. Lower urinary tract symptoms (LUTSs), which are highly prevalent in MS patients (affecting over 80% of patients), are closely intertwined with the location of the plaque, that is either intracranial or spinal. Even, in some cases, LUTSs are the primary manifestation of MS (in 10% of patients), and also patients’ disability status is usually related to the severity of their symptoms. Overactive bladder symptoms are the most frequently reported complaints. Urinary urgency (38%-99% of patients), increased urinary frequency (26%-82% of patients) and urge incontinence (27%-66% of patients), stress urinary incontinence (with a prevalence of 56%), and mixed urinary incontinence are among the mostly reported symptoms of patients with MS, which cause a significant decrease of QoL. By contrast, symptoms of the voiding phase are less frequent (6%-49%). Symptoms of both the storage and voiding phases can coexist in 50% of patients.^10^

Urinary tract is regulated by the medial prefrontal cortex, insula, and pons, and lesions in cortical regions lead to detrusor overactivity (DO). In addition, spinal cord, and particularly suprasacral lesions that are common in MS patients, may cause DO by impacting the descending inhibition of bladder contraction. Reticulospinal tract damage may lead to detrusor–sphincter–dyssynergia (DSD). Urinary retention may result from plaques that obstruct emptying in the efferent or afferent pathways. Only 5% of patients with sacral lesions have bladder areflexia, despite the fact that 63% of them exhibit detrusor hypocontractility.^39^

Litwiller et al, in a meta-analysis, showed that 62% of MS patients had Neurogenic detrusor overactivity (NDO). The other signs were DSD (25%), and Detrusor underactivity (DU) (20%). In addition, 10% were normal on examination. Low bladder capacity, increased PVR volume, and increased DO amplitude are common in MS patients. As MS is a fluctuating disease, and often presents in recurring attacks, in which the symptoms become worse or new symptoms appear, and between attacks, Urodynamic studies (UDS) shows urinary tract function at particular time points despite the fact that the symptoms may get better or stay the same.^3^

Different studies reported various prevalence of urinary symptoms in MS patients. A prevalence of 37%-99% for OAB, characterized by irritative bladder symptoms, 34%-79% for obstructive symptoms, and 25% for chronic urinary retention was reported.^6^

Management of MS-induced NLUTD requires a multidisciplinary model. Some of the most common approaches are medical therapies, such as antimuscarinics, intermittent self-catheterization, the use of synthetic antidiuretic hormone desmopressin, cannabinoids, and intravesical treatments like Onabotulinum toxin A (BoNTA) injection. Other therapeutic approaches are neuromodulation, including TNS and sacral nerve stimulation (SNS), and surgical treatments such as cystoplasty and non-continent urinary diversion.^10^

After behavioral therapies and medication management, nerve stimulation and neuromodulation is the third-line therapy used to treat these patients.^40^

Although the exact mechanism of TNS or sacral nerve root S3 stimulation in managing OAB remains uncertain, its efficacy has been proved. However, it is thought to be a result of modulation of spinal pelvic reflexes through the activation of inhibitory interneurons.^41^

The effects of TNS for NLUTD and OAB syndrome have also been the subject of multiple systematic reviews in the recent literature.^42-45^ However, just 1 systematic review that examined the impact of PTNS on MS-induced NLUTD in patients with MS was published, and the findings were favorable.^46^ The effects of TTNS on female MS patients with OAB syndrome have also been the subject of a protocol for a systematic review; however, its results have not yet been made public.^47^

The unique aspect of our study is the combination of these 2 approaches, which closes this knowledge gap and tackles both PTNS and TTNS in MS patients, offering fresh perspectives on the overall impacts of cutting-edge neuromodulation techniques.^48^

### Limitations

Despite providing level 1 evidence, this study may be subject to bias, primarily due to the inclusion of nonrandomized and uncontrolled trials with limited populations. Second¸ none of the studies addressed the long-term efficacy of the TNS; therefore, it is yet unknown whether the improvement of NLUTD is lifelong, and generalizing the results to clinical settings is rather restricted.

## Conclusion

The results of the current systematic review showed that stimulation of the tibial nerve shows a promising future in managing NLUTD in MS patients. However, due to the high heterogeneity among studies, these results must be interpreted with caution. The long-term effects of TNS therapy and its cost-effectiveness need to be addressed further by high-quality and controlled trials.

## Figures and Tables

**Table 1.** Characteristics of Included Studies

Citation	Origin	Design	Arms (n)	Patients					Intervention					F/U (Weeks)	Main Outcomes
				Number	Male/Female	Mean Age (Years)	EDSS Score	Duration of MS (Years)	Duration of NLUTD (Years)	Session/Week	Duration (Minutes)	Frequency	Pulse Width		
*PTNS*															
Fjorback (2007)	Denmark	Case series	1	12	7/5	46	–	–	–	–	–	20 Hz	200 μs	None	Detrusor overactivity via 2 successive slow-fill cystometries
Kabay (2008)	Turkey	Clinical trial	1	29	12/17	46.5 ± 8.5	4.8 ± 1.9	8.80 ± 3.6	4.3 ± 1.8	–	–	20 Hz	200 ms	None	Acute urodynamic effects, including mean first involuntary detrusor contractions and mean maximum cystometric capacity
Kabay (2009)	Turkey	Clinical trial	1	19	6/13	44.9 ± 8.3	4.6 ± 1.8	8.2 ± 3.3	3.9 ± 1.6	1	30	20 Hz	200 μs	12	Urodynamic findings including mean volume at the first involuntary detrusor contraction, mean maximum cystometric capacity, mean *P*_detmax_ at first involuntary detrusor contraction, maximal detrusor pressure at maximum cystometric capacity, detrusor pressure at maximal flow (*P*_detQmax_), and maximal flow rate (*Q*_max_)
Kabay (2015)	Turkey	Clinical trial	1	21	5/16	42.7 ± 6.9	4.2 ±1.5	9.56 ± 3.6	4.8 ±1.6	4-day intervals for 3 months, 21-day intervals for 3 months, and 28-day intervals for 3 months	–	20 Hz	200 μs	12	Voiding diary, ICIQ-SF, OAB-V8, and OAB-q SF
Gobbi (2011)	Switzerland	Clinical trial	1	18	2/16	46 ± 13	4.5 ± 2.1	15 ± 10	–	1	30	20 Hz	200 μs	12	3-Day frequency volume chart, PPBC questionnaire, PPIUS, patient assessment of urgency bother (VAS-UB), KHQ, and VAS score
Zecca (2014) (A)	UK	Cohort	1	83	21/62	49	4.1	14.4	–	1	30	20 Hz	200 μs	12	3-Day bladder diary, PPBC questionnaire, 7-level GRA scale, OAB-q, PPIUS, KHQ, and OAB-q
Zecca (2014) (B)	UK	Clinical trial	1	83	21/62	49	4	14.4	–	1	30	20 Hz	200 ms	NA	3-Day frequency volume chart and PPBC questionnaire, patient perception of VAS TS-VAS, 7-level GRA scale
*TTNS*															
De Seze (2011)	France	Clinical trial	1	70	19/51	48.3 ± 10.2	4.03 ± 1.60	13.4 ± 9.3	8.2 ± 7.8	7	30	10 Hz	200 μs	12	Clinical efficacy on urgency, based on warning time (WT), urgency “Mesure du Handicap Urinaire (MHU)” subscale, 3 days voiding chart, QoL, urodynamic parameter changes, and tolerance
Dunya (2020)	Turkey	Clinical trial	A_1_: TTNS A_2_: PFMT	55	All female	43.49 ± 8.66	3.69 ± 1.64	13.70 ± 8.63	7.67 ± 6	5	30	10 Hz	200 μs	6	Bladder diary, PVR, and Qualiveen scales
Dunya (2021)	Turkey	Clinical trial	A_1_: TTNS A_2_: PFMT	30	All female	42.80 ± 7.74	3.40 ± 1.52	13.70 ± 9.40	7.42 ± 6.15	5	30	10 Hz	200 ms	6	Sexual function index (FSFI), OABv‐8, and sexual quality of life—female (SQoL‐F) questionnaires
Al Dandan (2021)	Ireland	Clinical trial	1	20	6/14	–	–	–	–	3	30	10 Hz	0.2-0.5 ms equal to 200 μs	6	Feasibility of the intervention, bladder storage symptoms, and quality of life using ICIQ-OAB
Zonić-Imamović (2019)	Bosnia and Herzegovina	Randomized clinical trial	A_1_: TTNS A_2_: oxybutynin	60	20/40	A_1_: 47.36 ± 7.98 A_2_: 45.8 ± 8.13	–	A_1_: 8.9 ± 4.9 A_2_: 8.3 ± 5.1	–	7	30	10 Hz	200 ms	12	OAB-q SF, QoF

EDSS, Expanded Disability Status Scale; F/U, follow-up; ICIQ-OAB, International Consultation on Incontinence Questionnaire on OAB; KHQ, King’s Health Questionnaire; MS, multiple sclerosis; NLUTD, neurogenic lower urinary tract dysfunction; PFMT, pelvic floor muscle training; PPBC, patient perception of bladder condition; PPIUS, Patient Perception of Intensity of Urgency Scale; PTNS, percutaneous tibial nerve stimulation; PVR, post-voiding residue; QoL, Quality of Life; SF, Short Form; TS-VAS, treatment satisfaction via using a visual analog scale; TTNS, transcutaneous tibial nerve stimulation; VAS, visual analog scale.

**Table 2.** The Assessment of Included Studies Based on the JBI Checklist

Citation	Q_1_	Q_2_	Q_3_	Q_4_	Q_5_	Q_6_	Q_7_	Q_8_	Q_9_	Q_10_	Q_11_	Q_12_	Q_13_	Quality of the study
*Case series*														
Fjorback (2007)	Yes	Yes	Yes	No	No	No	No	Yes	No	Yes	–	–	–	Low
*Cohort*														
Zecca (2014) (A)	Yes	Yes	Yes	No	No	No	Yes	Yes	Yes	Not applicable	Yes	–	–	Moderate
*Nonrandomized clinical trial*														
Kabay (2008)	Yes	Yes	Yes	No	Yes	Not applicable	Yes	Yes	Yes	–	–	–	–	Moderate
Kabay (2009)	Yes	Yes	Yes	No	Yes	Yes	Yes	Yes	Yes	–	–	–	–	High
Kabay (2015)	Yes	Yes	Yes	No	Yes	Yes	Yes	Yes	Yes	–	–	–	–	High
Gobbi (2011)	Yes	Yes	Yes	No	Yes	Yes	Yes	Yes	Yes	–	–	–	–	High
Zecca (2014) (B)	Yes	Yes	Yes	Yes	Yes	Not applicable	Yes	Yes	Yes	–	–	–	–	High
De Seze (2011)	Yes	Yes	Yes	No	Yes	Yes	Yes	Yes	Yes	–	–	–	–	High
Dunya (2020)	Yes	Yes	Yes	No	Yes	Yes	Yes	Yes	Yes	–	–	–	–	High
Dunya (2021)	Yes	Yes	Yes	No	Yes	Yes	Yes	Yes	Yes	–	–	–	–	High
Al Dandan (2021)	Yes	Yes	Yes	No	Yes	Yes	Yes	Yes	Yes	–	–	–	–	High
*Randomized clinical trial*														
Zonić-Imamović (2019)	Unclear	Unclear	Yes	No	Unclear	Unclear	Yes	Yes	Yes	Yes	Yes	Yes	No	Low

JBI, Joanna Briggs Institute.

**Table 3.** The Results of Meta-analysis

Groups	Outcome	Effect Size and 95% CI					Test of Null (2-Tail)		Heterogeneity			
		Number of Studies	Point Estimate	Standard Error	Lower Limit	Upper Limit	*Z*-Value	*P*	*Q*-Value	df (*Q*)	*P*	*I* ^2^
PTNS	Frequency	3	–2.991	0.801	–4.561	–1.421	–3.733	.000	36.521	2	.00	94.524
TTNS			3	–2.191	0.370	–2.916	–1.466	–5.922	.000	0.215	2	.90	0.000
			6	–2.623	0.491	–3.585	–1.661	–5.343	.00	38.589	5	.00	87.043
PTNS	PVR	3	–45.478	7.843	–60.849	–30.107	–5.799	.000	0.789	2	.67	0.000
TTNS			2	–14.301	3.325	–20.817	–7.784	–4.301	.000	0.007	1	.93	0.000
Total			5	–31.132	9.946	–50.625	–11.638	–3.130	.002	14.193	4	.01	71.816
PTNS	Urgency	1	–7.280	0.567	–8.392	–6.168	–12.831	.00	0.000	0	1.00	0.000
TTNS			2	–3.303	0.659	–4.594	–2.011	–5.012	.00	1.294	1	.26	22.749
Total			3	–4.696	1.504	–7.643	–1.749	–3.123	.00	25.516	2	.00	92.162
PTNS	MCC	2	80.941	12.676	56.096	105.785	6.385	.00	10.274	1	.00	90.267
TTNS			1	38.700	18.539	2.364	75.036	2.087	.04	0.000	0	1.00	0.000
Total			3	70.957	13.399	44.694	97.219	5.296	.00	18.252	2	.00	89.042
PTNS	MVV	3	75.011	18.165	39.409	110.614	4.129	.00	13.371	2	.00	85.043
PTNS	Nocturia	3	–2.036	0.189	–2.407	–1.665	–10.750	.00	5.221	2	.07	61.696
TTNS			2	–0.392	0.390	–1.156	0.373	–1.005	.315	6.257	1	.01	84.018
Total			5	–1.419	0.413	–2.229	–0.609	–3.434	.00	82.539	4	.00	95.154
PTNS	Urinary incontinence	1	–3.330	0.391	–4.096	–2.564	–8.525	.00	0.000	0	1.00	0.000
TTNS			3	–1.464	0.959	–3.344	0.416	–1.527	.127	15.023	2	.00	86.687
Total			4	–2.000	1.051	–4.061	0.060	–1.903	.057	62.842	3	.00	95.226

**Supplementary Table 1. T1675248171000:** Characteristics of Studies with Mixed Populations

TTNS							
Welk, 2020	Canada	RCT	OAB+neurogenic bladder	50 (not mentioned)	10/40	62	Patient perception of bladder condition (PPBC)
Amarenco, 2004	France	Prospective, open-label trial	Neurologic+idiopathic	44 (13)	15/29	53.3	Urodynamic parameters including mean first involuntary detrusor contraction volume, maximum cystometric capacity
Seth, 2018	UK	Prospective, single centre, open label	Neurologic+idiopathic	48 (24)	10/38	46.4 and 46.9 in two arms	ICIQ-OAB,ICIQ-LUTSqol , 3-day bladder diary and a Global Response Assessment (GRA)
Valles-Antuna, 2017	Spain	prospective cohort	Urge urinary incontinence (UUI)	65 (9)	24/41	55.06	48-hour micturitional calendar
Tornic, 2019	Switzerland	RCT	Neurogenic LUTD	9 (2 )	7/2	52.8	feasibility, acceptability, safety of TTNS
PTNS							
Tudor, 2018	UK	retrospective cohort	Neurologic+idiopathic	74 (19 )	22/52	56	ICIQ-OAB, ICIQ-LUTSqol, 3-day bladder diary parameters
Jung 2020, (A)	USA	retrospective cohort	OAB	141 (not mentioned)	All female	70	PGI-I, OABq-SF
Jung 2020, (B)	USA	retrospective cohort	OAB	334 (not mentioned)	All female	70.9	PGI-I, OABq-SF
Salatzki, 2019	UK	cross-sectional	OAB	79 (27)	20/59	58.9	bladder diary, PTNS Service Evaluation Questionnaire (PTNS-SEQ), ICIQ-OAB, ICIQ-LUTSqol
Arrabal-Polo, 2012	Spain	prospective cohort	OAB	14 (1)	All female	60.8	48-hour micturitional calendar

1. F/U: Follow-up; 2. MS: Multiple sclerosis; 3. M/F: Male/female; 4. TTNS: Transcutaneous tibial nerve stimulation; 5. RCT: Randomized controlled trial; 6. OAB: Overactive bladder; 7. Hz: Hertz; 8. ms: Millisecond; 9. ICIQ-OAB: International Consultation on Incontinence Questionnaire on OAB; 10. ICIQ-LUTSqol: International Consultation on Incontinence Questionnaire Lower Urinary Tract Symptoms Quality of Life Module; 11. LUTD: Lower urinary tract dysfunction; 12. μs: Microsecond; 12. PGI-I: Patient Global Impression- Improvement; 12. OABq-SF: Overactive Bladder Questionnaire-Short Form
